# Novel Pathogenic Variants in the Gene Encoding Stereocilin (*STRC*) Causing Non-Syndromic Moderate Hearing Loss in Spanish and Argentinean Subjects

**DOI:** 10.3390/biomedicines11112943

**Published:** 2023-10-31

**Authors:** María Domínguez-Ruiz, Laura Ruiz-Palmero, Paula I. Buonfiglio, Irene García-Vaquero, Elena Gómez-Rosas, Marina Goñi, Manuela Villamar, Matías Morín, Miguel A. Moreno-Pelayo, Ana B. Elgoyhen, Francisco J. del Castillo, Viviana Dalamón, Ignacio del Castillo

**Affiliations:** 1 Servicio de Genética, Hospital Universitario Ramón y Cajal, 28034 Madrid, Spain; mdominguezr@salud.madrid.org (M.D.-R.); lruizpalmero@gmail.com (L.R.-P.); igarciavaquero@salud.madrid.org (I.G.-V.); elenagomez_tel@yahoo.es (E.G.-R.); marinagoni13@hotmail.com (M.G.); manuela.villamar@salud.madrid.org (M.V.); matmorinro@yahoo.es (M.M.); mmorenop@salud.madrid.org (M.A.M.-P.); fcastillo@salud.madrid.org (F.J.d.C.); 2 Centro de Investigación Biomédica en Red de Enfermedades Raras (CIBERER), 28034 Madrid, Spain; 3 Laboratory of Physiology and Genetics of Hearing, Instituto de Investigaciones en Ingeniería Genética y Biología Molecular “Dr. Héctor N. Torres”, Consejo Nacional de Investigaciones Científicas y Técnicas, Vuelta de Obligado 2490, Ciudad Autónoma de Buenos Aires C1428ADN, Argentina; paulabuonfiglio@gmail.com (P.I.B.); abelgoyhen@gmail.com (A.B.E.); 4 Instituto de Farmacología, Facultad de Medicina, Universidad de Buenos Aires, Ciudad Autónoma de Buenos Aires C1121ABG, Argentina

**Keywords:** non-syndromic hearing impairment, DFNB16, *STRC*, stereocilin, genetic epidemiology

## Abstract

Non-syndromic hearing impairment (NSHI) is a very heterogeneous genetic condition, involving over 130 genes. Mutations in *GJB2*, encoding connexin-26, are a major cause of NSHI (the DFNB1 type), but few other genes have significant epidemiological contributions. Mutations in the *STRC* gene result in the DFNB16 type of autosomal recessive NSHI, a common cause of moderate hearing loss. *STRC* is located in a tandem duplicated region that includes the *STRCP1* pseudogene, and so it is prone to rearrangements causing structural variations. Firstly, we screened a cohort of 122 Spanish familial cases of non-DFNB1 NSHI with at least two affected siblings and unaffected parents, and with different degrees of hearing loss (mild to profound). Secondly, we screened a cohort of 64 Spanish sporadic non-DFNB1 cases, and a cohort of 35 Argentinean non-DFNB1 cases, all of them with moderate hearing loss. Amplification of marker D15S784, massively parallel DNA sequencing, multiplex ligation-dependent probe amplification and long-range gene-specific PCR followed by Sanger sequencing were used to search and confirm single-nucleotide variants (SNVs) and deletions involving *STRC*. Causative variants were found in 13 Spanish familial cases (10.7%), 5 Spanish simplex cases (7.8%) and 2 Argentinean cases (5.7%). In all, 34 deleted alleles and 6 SNVs, 5 of which are novel. All affected subjects had moderate hearing impairment. Our results further support this strong genotype–phenotype correlation and highlight the significant contribution of *STRC* mutations to moderate NSHI in the Spanish population.

## 1. Introduction

Hearing impairment exerts a strong impact on the lives of the affected subjects. In developed countries, the majority of cases have a genetic etiology [1]. Non-syndromic hearing impairment (NSHI), in which the hearing loss is not associated with clinical signs in other organs, accounts for 70% of the genetic cases and it is a very heterogeneous condition, with over 130 genes known to be involved to date [2]. Up to 80% of NSHI cases follow an autosomal recessive pattern of inheritance.

Mutations in *GJB2*, encoding connexin-26, are a major cause of NSHI (the DFNB1 type, accounting for up to 50% of the cases). Few other genes have significant epidemiological contributions. Remarkably, mutations in the *STRC* gene, which result in the DFNB16 type of autosomal recessive NSHI [3], are frequently found in subjects with moderate hearing loss, and they account for 11–16% of genetically elucidated non-DFNB1 cases in different populations [4,5]. 

The *STRC* gene encodes stereocilin, a protein which locates to the stereocilia bundle of outer hair cells in the inner ear. Specifically, it is a component of the horizontal top connectors, which link stereocilia to each other, and also a component of the attachment crowns that couple the tallest stereocilia to the tectorial membrane [6,7,8]. The absence of these two structures is responsible for the moderate hearing loss that is observed in DFNB16 affected subjects.

**Figure 1:**
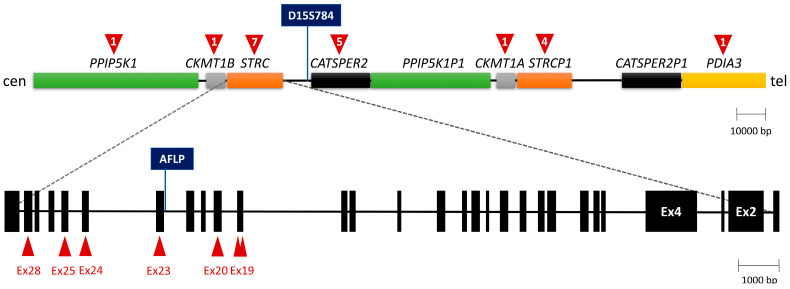
Tandemly duplicated region that contains the *STRC* gene on 15q15.3. Above: the number of MLPA probes is indicated within the red arrowhead above each gene. Below: exon–intron structure of the *STRC* gene, red arrowheads indicating the position of the MLPA probes on different exons.

*STRC* is located on 15q15.3, in a tandemly duplicated region that contains four genes (Figure 1). The functional *STRC* is located in the proximal repeat, whereas the distal duplication contains its non-processed pseudogene (*STRCP1*). Both gene and pseudogene contain 29 exons, with a 100% identity between them for exons 1 to 15. In contrast, there are a few sequence variants between *STRC* and *STRCP1* from exon 16 to exon 29, which allow for discrimination between gene and pseudogene. This duplicated structure makes it prone to rearrangements, so that large deletions are the most common type of *STRC* mutation, with a carrier frequency of up to 1.36% in studied populations [4]. In some individuals, the deletion extends to encompass the adjacent *CATSPER2* gene, resulting in the deafness-infertility syndrome in homozygous males [9].

The analysis of *STRC* has been challenging because of the extremely high identity between gene and pseudogene. An early approach for detecting deletions was the amplification of the non-polymorphic D15S784 marker, which is located between *STRC* and *CATSPER2* and it is absent from the distal repeat (Figure 1) [9]. This technique proved effective for many families with deletions of both *STRC* alleles. More recently, Multiplex Ligation-dependent Probe Amplification (MLPA) is being used to detect or confirm deletions in exons 19 to 28, for which specific probes are available. Moreover, massively parallel DNA sequencing is able to identify Copy Number Variations (CNVs) and Single-Nucleotide Variants (SNVs) in the *STRC* gene. Subsequently, long-range gene-specific PCR followed by Sanger sequencing is needed to confirm that a SNV is located in the gene and not in the pseudogene.

In this work, we have used these diverse experimental approaches to analyze several cohorts of hearing-impaired subjects from Spain and Argentina. We identified causative *STRC* variants in 20 unrelated cases, all of whom exhibited moderate hearing impairment. Our results further support this solid genotype–phenotype correlation and the significant contribution of *STRC* pathogenic variants to moderate NSHI in all studied populations.

## 2. Materials and Methods

### 2.1. Human Subjects

We enrolled a cohort of 221 Spanish unrelated familial cases of autosomal recessive NSHI. All cases in this cohort had at least two affected siblings with unaffected parents. Before this work, a preliminary screening of the *GJB2* gene (coding region and splice sites) by Sanger sequencing, as well as testing for the common del(*GJB6*-D13S1830) and del(*GJB6*-D13S1854) deletions in the DFNB1 locus, revealed causative genetic variants in 99 families. The remaining 122 families were investigated in this study. In this cohort, the degree of hearing loss was profound in 25 cases, severe in 18 cases, moderate in 75 cases and mild in 4 cases. 

Subsequently, we also included in this study a cohort of 64 Spanish simplex non-DFNB1 cases, and a cohort of 35 Argentinean non-DFNB1 cases (3 familial and 32 simplex). All affected subjects in these two cohorts had moderate hearing loss. A diagram of the study setup is shown in Figure S1.

Hearing was evaluated by pure-tone audiometry, testing for air conduction (frequencies 250–8000 Hz) and bone conduction (frequencies 250–4000 Hz). Hearing impairment was classified as mild (21–40 dB HL), moderate (41–70 dB HL), severe (71–95 dB HL) and profound (>95 dB HL), according to the pure tone average (PTA) threshold levels at 0.5, 1, 2 and 4 kHz.

### 2.2. DNA Purification, Genotyping and MLPA

DNA was extracted from peripheral blood samples by using the Chemagic Magnetic Separation Module I automated system (Chemagen, Baesweiler, Germany).

Microsatellite markers D15S780, D15S778, D15S1039 and D15S126 were amplified using fluorescently labeled primers and PCR conditions as previously reported [10,11]. Amplified alleles were resolved by capillary electrophoresis in an ABI Prism 3100 Avant Genetic Analyzer (Applied Biosystems, Waltham, MA, USA).

Amplification of marker D15S784 (upper primer 5′- GAGACCCTGTCTCAAAACAC -3′, lower primer 5′-GAGGAATGACTGAAATGATTT-3′) was performed through a multiplex PCR that includes an amplification control (exon 4 of *GJB6*: upper primer 5′- CGTCTTTGGGGGTGTTGCTT-3′, lower primer 5′-CATGAAGAGGGCGTACAAGTTAGAA-3′). Two bands are expected in normal individuals: a 168 bp band corresponding to the D15S784 marker (absent in biallelic deletions) and a 333 bp band corresponding to exon 4 of *GJB6*.

The rationale of the AFLP (Amplified Fragment Length Polymorphism) assay is reported in Section 3.1. PCR conditions: upper primer, 5′-TGGCATCTCCAATCACTTCTTGTTC-3′; lower primer, 5′-AATGTCCCTCGTACATCTGCACAA-3′; annealing temperature, 63 °C.

The MLPA P461 kit (MRC-Holland, Amsterdam, The Netherlands) was used to detect copy number variations in the DFNB16 locus. It includes probes for exons 19, 20, 23, 24, 25 and 28 of *STRC*, and for its neighboring genes (Figure 1).

### 2.3. Sanger DNA Sequencing

For *STRC*-specific amplification, we developed two long-range PCR assays that generate two overlapping amplicons that cover the complete *STRC* sequence (PCR1, from upstream exon 1 to intron 22; and PCR2, from intron 18 to downstream of exon 29). To achieve specificity, primers were located on sequence stretches containing unique SNPs within the gene (Table 1). We used Takara LA Taq polymerase (Takara Bio, Shiga, Japan) according to the manufacturer’s protocol, with an extension time of 10 min and 45 s for PCR1, and 6 min and 30 s for PCR2. 

Sanger sequencing was performed exon by exon using these two amplicons as templates, in a 1/20 dilution. Primers to amplify *STRC* exons and their flanking intronic sequences are shown in Table S1. We did not find any sequence variant corresponding to *STRCP1*, which confirmed *STRC*-specificity.

### 2.4. Targeted Massively Parallel DNA Sequencing

We used the OTO-NGS v2 gene panel, which was developed in our laboratory. It includes 117 genes that are known to be involved in NSHI, and it is based on the IDT probes capture system [12]. Captured enriched-libraries were sequenced on the Illumina NextSeq 550 platform (Illumina, Inc., San Diego, CA, USA). Sequence data were mapped against human genome GRCh37/hg19 reference sequence and analyzed using Sophia Genetics’ software v5.10.42.1 (Sophia Genetics, Rolle, Switzerland) to annotate and prioritize SNVs and CNVs.

### 2.5. Assessment of Pathogenicity of DNA Variants

Pathogenicity of DNA variants was assessed according to the guidelines from the American College of Medical Genetics and Genomics and the Association for MolecularPathology (ACMG/AMP) [13], as implemented by Varsome [14], using GRCh38 as human reference genome. Scores were subsequently modified manually to delete criterion PP2 and to take into consideration criterion PM3, as recommended in the disease-specific ACMG/AMP guidelines for hearing loss [15].

## 3. Results

### 3.1. Haplotype Analysis and PCR-Based Detection of Deletions Involving STRC

We studied a cohort of 122 Spanish familial cases of autosomal recessive NSHI, with at least two affected siblings and unaffected parents, in whom DFNB1 pathogenic variants had been previously excluded. A first subcohort of 37 of these 122 familial cases was investigated before the advent of massively parallel DNA sequencing. All siblings in every family and their parents were genotyped for four polymorphic microsatellite markers that are closely linked to *STRC* on 15q15.3 (order of markers: cen-D15S780-D15S778-*STRC*-D15S1039-D15S126-tel). In 13 of the 37 families, haplotype analysis could not exclude genetic linkage to DFNB16 (Figure S2). They were further investigated through a combination of techniques, as follows.

Firstly, we performed PCR amplification of the non-polymorphic D15S784 marker, as a tool to detect deletions in the region (Figure 1) [9]. In 5 of the 13 families that were tested (HRC1, HRC3-HRC6), the D15S784 marker could not be amplified, suggesting deletion of both alleles of the *STRC* gene.

Since the D15S784 marker is not located within *STRC*, we performed a complementary test, which takes advantage of the fact that there is a 31 bp intronic sequence located 28 bp upstream from *STRC* exon 23, which is not present in the pseudogene. We designed an AFLP assay so that PCR amplification of a DNA segment containing this variant results in two bands of different sizes (228 bp for the gene and 197 bp for the pseudogene). In patients with deletion of both alleles of *STRC*, only the small band corresponding to the pseudogene is obtained (Figure S3). This test was performed on the five families that did not amplify the D15S784 marker, confirming that the deletion affected *STRC*.

Furthermore, Multiplex Ligation-dependent Probe Amplification (MLPA) was performed on probands from the 13 families in which genetic linkage to DFNB16 had not been excluded, to confirm the deletions in the probands from families HRC1 and HRC3-HRC6, to investigate hypothetical heterozygous deletions in the remaining cases, and to study the segregation of the deletions in parents and unaffected siblings. All biallelic deletions were confirmed in the affected subjects, their parents being heterozygous. Moreover, a heterozygous deletion was detected in the proband of family HRC2, which he had inherited from his father (Figure S2). In the 11 deletions, all MLPA probes on *CKMT1B* (1 probe), *STRC* (7 probes) and *CATSPER2* (5 probes) detected zero copies, whereas the probe on *PPIP5K1* and the probe on *CKMT1A* detected two copies (Figure 1).

Given the complete sequence identity between *STRC* and *STRCP1* for exons 1–15, it is not possible to screen the gene for SNVs through sequencing exon by exon directly from genomic DNA. To circumvent this problem, we developed two gene-specific PCR assays, which generate two overlapping amplicons that cover the complete *STRC* sequence (exon 1 to intron 22, and intron 18 to exon 29, respectively). Sanger sequencing was performed exon by exon on these two amplicons from the proband of family HRC2. This approach revealed the novel c.4483_4484del, p.(Phe1495Cysfs*9) variant in exon 23 of *STRC* (Figure S4). The patient was compound heterozygous for a deletion and this pathogenic SNV, which he had inherited from his mother. Segregation of all pathogenic variants that were found in families HRC1 to HRC6 is shown in Figure S2.

The same sequencing strategy was used to screen the remaining seven cases in which genetic linkage to DFNB16 had not been excluded. No pathogenic variant in *STRC* was found in any of these cases. Other studies that were being performed in parallel revealed causative variants in DFNB genes other than *STRC* in three of these seven cases.

In parallel, a cohort of 35 unrelated Argentinean non-DFNB1 cases (3 familial and 32 simplex) with moderate NSHI was screened for deletions in the DFNB16 locus, through the PCR-based D15S784 test, followed by MLPA. In simplex case IHT2, both alleles of *STRC* were absent, the deletion encompassing *CKMT1B*, *STRC* and *CATSPER2* (all 13 MLPA probes in these genes detected zero copies). In familial case IHT1, a heterozygous deletion encompassing only *STRC* was found. Further analysis through Sanger sequencing on the two *STRC*-specific amplicons revealed a novel nonsense variant, c.1030C > T, p.(Arg344*) in exon 4. The two affected subjects in the family were compound heterozygous for this pathogenic SNV and the deletion.

### 3.2. Screening through Massively Parallel DNA Sequencing

Massively parallel DNA sequencing of a targeted gene panel containing 117 genes that are known to be involved in NSHI was used to investigate a second subcohort of 85 out of the 122 Spanish familial cases (see above), and a cohort of 64 Spanish simplex cases with moderate NSHI. Sequence variants identified through the gene panel were confirmed and their segregation was studied using MLPA and *STRC*-specific Sanger sequencing (Figure S4). We elucidated 12 unrelated cases. Nine of these cases (four familial and five simplex) have complete or partial deletions of both alleles of *STRC*, two familial cases are compound heterozygous for a SNV and a deletion, and one familial case is compound heterozygous for two SNVs in *STRC* (Table 2). In all cases, parents were heterozygous carriers as expected.

Three of these four SNVs were novel. These included two nonsense variants (p.(Glu1275*) and p.(Gly1366*), in exons 19 and 20, respectively) and two missense variants: p.(Pro1520Arg) in exon 24, previously reported [16], and the novel p.(Cys590Arg), in exon 4. It results in a radical replacement of a highly conserved residue in the protein (Figure S5) and it was classified as “likely pathogenic” according to the ACMG/AMP criteria (PM2 supporting, PM3 and PP1 moderate, PP3) [13,14,15]. 

### 3.3. Genotype–Phenotype Correlations

**Figure 2:**
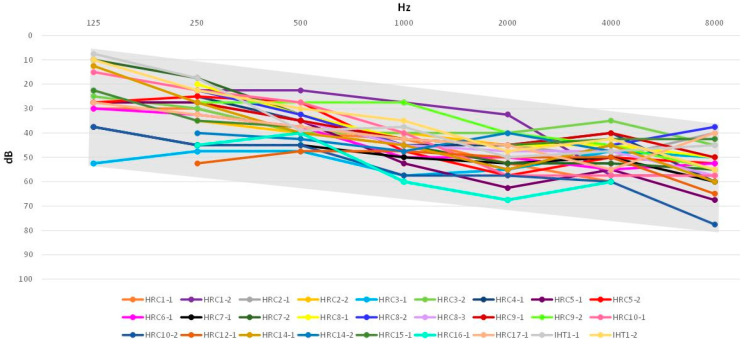
Accumulated audiograms of 27 subjects with biallelic *STRC* pathogenic variants. Only results for air conduction are shown. As the hearing loss was bilateral and symmetrical, mean values of the two ears are shown for each patient.

We obtained audiological data from 27 affected subjects from the 20 families with biallelic pathogenic variants in *STRC*. All of them present with moderate hearing impairment (Figure 2). Onset of the hearing loss occurred in early childhood (age range 0–8 years) in a majority of cases (73.5%), and in the 9–18 years-old range in the remaining cases, although this apparently delayed onset may reflect a delay in diagnosis (Figure S6). 

## 4. Discussion

Since the discovery of its involvement in autosomal recessive NSHI in 2001 [3], screening *STRC* for causative variants has been hampered by the structure of the duplicated region where the gene lies. Moreover, the 100% identity between *STRC* and *STRCP1* across the 5′ half of their respective sequences has complicated the search for SNVs by Sanger sequencing [3,17]. Different approaches have been used to circumvent these problems [17,18,19,20,21]. Here we provide some tools to facilitate these screenings: an AFLP test to quickly detect large biallelic deletions involving *STRC*, and two long-range PCR assays (PCR1 and PCR2), which generate *STRC*-specific templates on which Sanger sequencing can be performed exon by exon. Even in these times of massively parallel sequencing, it is still needed to confirm whether the sequence variants that are found are really located in the gene and not in the pseudogene. In fact, many SNVs in the literature have not been confirmed to be *STRC*-specific [18]. 

In several studies on large cohorts of hearing-impaired subjects who were investigated specifically for *STRC* variants, DFNB16 has been considered a major cause of NSHI, being second in frequency only to DFNB1 in most tested populations [4,17,19,22,23,24]. This conclusion has received further support from screenings of large cohorts of hearing-impaired subjects through massively parallel sequencing of targeted gene panels or exomes [5], and references therein]. In a systematic review and meta-analysis study, the DFNB16 prevalence in non-DFNB1 genetically elucidated patients was found to be 11.1% [4]. In this work, on patients referred to the Service of Genetics of Hospital Ramón y Cajal over the years, 13 out of 122 (10.7%) Spanish familial cases with non-DFNB1 autosomal recessive NSHI and 6 out of 64 (9.4%) Spanish simplex cases with non-DFNB1 moderate NSHI were shown to carry biallelic pathogenic variants in *STRC*, two figures that are very close to that of the meta-analysis. These results confirm DFNB16 as a major contributor to NSHI also in the Spanish population. In Argentinean cases, 2 out of 35 cases (5.7%) carried biallelic pathogenic variants in *STRC*. This apparently lower frequency is likely a consequence of the cohort that was tested, smaller in size and predominantly composed of simplex cases (32 out of 35). In fact, one of the positive cases was familial. Anyway, comparison of the prevalence of the diverse types of NSHI across different populations is hampered by the variety of methodological approaches that are used for the screenings, and by the heterogeneity in size and composition of the cohorts (familial versus simplex cases, degree of hearing impairment, etc.) [5]. 

The tandemly duplicated structure of the region on 15q15.3 that contains, among others, the *STRC* gene, makes it prone to structural rearrangements, that are thought to be generated by non-allelic homologous recombination. Consequently, the most frequent type of pathogenic variants are deletions, which may account for about 86% of the mutant alleles [21]. In our work, deletions account for 34 of 40 (85%) of the mutant alleles, in agreement with the previous study. We have classified the deletions that were found in our study in four subtypes according to the number of MLPA probes that were detected at zero copies. The most frequent type encompasses all probes on *CKMT1B*, *STRC* and *CATSPER2* (30 out of 34, 88.2%). In another study, 77% of the deletions belonged to this type [22]. This is relevant, as the concomitant removal of *STRC* and *CATSPER2* results in the deafness and male infertility syndrome. Eleven hearing impaired subjects of our cohort who carry biallelic type 1 deletions are males, a fact to be considered when providing genetic counseling and clinical follow-up.

Although we have grouped in a same type all deletions lacking the same MLPA probes, it should be noted that it does not mean that they are identical and patients are homozygous. The recombination events that gave rise to each deletion could have taken place in any position within the duplicated region, as it is likely that they occur recurrently. In fact, our analysis on families HRC1-HRC6 with type 1 deletions show that all of them are associated with different haplotypes (Figure S2). In a study using allele-specific droplet digital PCR, it was estimated that 37% of the cases who carried biallelic deletions were in fact compound heterozygous for two different deletions [21]. In our study we have identified one similar case (HRC15) (Table 2).

The DFNB16 hearing impairment is considered to be moderate, with a characteristic slightly down-sloping profile in the audiogram [4,17,18,19,20,21,22,23,24,25,26,27]. All the subjects with biallelic *STRC* pathogenic variants of our study from whom we could obtain audiological data fit within this model (Figure 2). In some studies, mild age-associated progression of the hearing loss was reported [20,27], and it was estimated in 0.6 dB per year [27]. A few patients in our study referred subjective progression of their hearing losses, but this could not be proved by examining their available audiograms. However, it must be noted that most of them are still young, and progression may be detected only after longer periods of time. As regards the age of onset, most studies report it as congenital [19,23,26] or in early childhood [17,18,20,21,22,24,25,26]. In our series, it was detected postnatally in 8 out of 27 subjects, and in early childhood in other 15 subjects (in all, 23 of 27, 85%) (Figure S6). Cases with apparently delayed onset may just reflect delayed diagnoses. In fact, we have observed earlier referred onsets in the second affected child of the same family. 

Finally, two recent studies have revealed that some DFNB16 hearing-impaired subjects present with recurrent benign paroxysmal positional vertigo [28,29]. In one of the studies, this clinical sign was found in 29 out of 64 (45.3%) DFNB16 cases [29]. None of our patients complained of vestibular dysfunction but, because of its episodic nature, it could have been overlooked, as in most other cases in the literature. A retrospective study would be needed to reevaluate this novel clinical sign that extends the DFNB16 phenotype.

## 5. Conclusions

The tools and screening strategies we have developed and used in this study are of help to the routine diagnosis of DFNB16 NSHI, as the region where the *STRC* gene lies poses special problems to molecular testing. Stratifying the patients according to the different genetic etiologies of NSHI is the first step towards the application of specific treatments, such as gene therapy, which are being investigated and will be implemented in a near future [30]. 

## Figures and Tables

**Table 1:** Primers and amplicon sizes for *STRC*-specific amplification.

Assay	Amplicon Size (bp)	Primers (5′-3′)
PCR1	15,665	Upper: AGTTTGTTTCTCCTGGGCGTCAT Lower: GAGCACTGTGAGAAATAGGGATCAAA
PCR2	6240	Upper: GCCCAGCTCCACCTGAATCC Lower. TGTGAACGGCGTCTGGAGAGA

**Table 2:** Genotypes of the affected subjects with pathogenic variants in *STRC*.

Case	Type	Allele 1	Allele 2
HRC1	F	Deletion—Type 1	Deletion—Type 1
HRC2	F	c.4483_4484del/p.(Phe1495Cysfs*9)	Deletion—Type 1
HRC3	F	Deletion—Type 1	Deletion—Type 1
HRC4	F	Deletion—Type 1	Deletion—Type 1
HRC5	F	Deletion—Type 1	Deletion—Type 1
HRC6	F	Deletion—Type 1	Deletion—Type 1
HRC7	F	c.3823G > T/p.(Glu1275*)	c.4559C > G/p.(Pro1520Arg) [16]
HRC8	F	Deletion—Type 1	Deletion—Type 1
HRC9	F	**c.1768T > C/p.(Cys590Arg)**	Deletion—Type 1
HRC10	F	Deletion—Type 2	Deletion—Type 2
HRC11	F	Deletion—Type 1	Deletion—Type 1
HRC12	S	Deletion—Type 1	Deletion—Type 1
HRC13	F	c.4096G > T/p.(Gly1366*)	Deletion—Type 1
HRC14	F	—Deletion—Type 1	Deletion—Type 1
HRC15	S	Deletion—Type 1	Deletion—Type 4
HRC16	S	Deletion—Type 1	Deletion—Type 1
HRC17	S	Deletion—Type 1	Deletion—Type 1
HRC18	S	Deletion—Type 1	Deletion—Type 1
IHT1	F	c.1030C > T/p.(Arg344*)	Deletion—Type 3
IHT2	S	Deletion—Type 1	Deletion—Type 1

Novel SNVs are indicated in bold. All of them were absent from gnomAD v2.1.1 and v3.1.2 databases, except c.1030C > T, which was found in gnomAD v2.1.1 with a highest population MAF (minor allele frequency) of 0.0001666. Reference cDNA sequence: NM_153700.2. F, familial; S, simplex. Deletions: Type 1: all 13 probes on *CKMT1B*, *STRC* and *CATSPER2* at zero copies. Type 2: all 8 probes on *CKMT1B* and *STRC* at zero copies. Type 3: all 7 probes on *STRC* at zero copies. Type 4: five probes (Ex19-Ex24) on *STRC* at zero copies.

## Data Availability

Data on the novel pathogenic variants that are reported in this study are available in ClinVar (accession numbers SCV004041561 to SCV004041565).

## Supplementary Materials

The following supporting information can be downloaded at: https://www.mdpi.com/article/10.3390/biomedicines11112943/s1, Figure S1: Diagram of the study setup; Figure S2: Pedigrees of the six families with biallelic pathogenic variants in the *STRC* gene for which haplotype analysis was performed; Figure S3: Example of AFLP test for the 3′ end of intron 22 in *STRC* and *STRCP1*; Figure S4: Electropherograms of the pathogenic SNVs in *STRC* that were found in this study; Figure S5: Alignment of estereocilin orthologous sequences from human and three other mammals in the stretch containing the residue that is affected by the p.(Cys590Arg) variant; Figure S6: Age of onset of the hearing loss in 27 subjects with biallelic pathogenic variants in *STRC*. Table S1: Primer sequences for Sanger sequencing of the *STRC* gene.
