# Supplementary material for: Novel Pathogenic Variants in the Gene Encoding Stereocilin (STRC) Causing Non-Syndromic Moderate Hearing Loss in Spanish and Argentinean Subjects
Source: Biomedicines. 2023 Oct 31;11(11):2943. doi: 10.3390/biomedicines11112943 (PMC10668944; doi:10.3390/biomedicines11112943)
Supplement: Supplementary file 1 [file biomedicines-11-02943-s001.zip › biomedicines-2683364-supplementary.pdf]

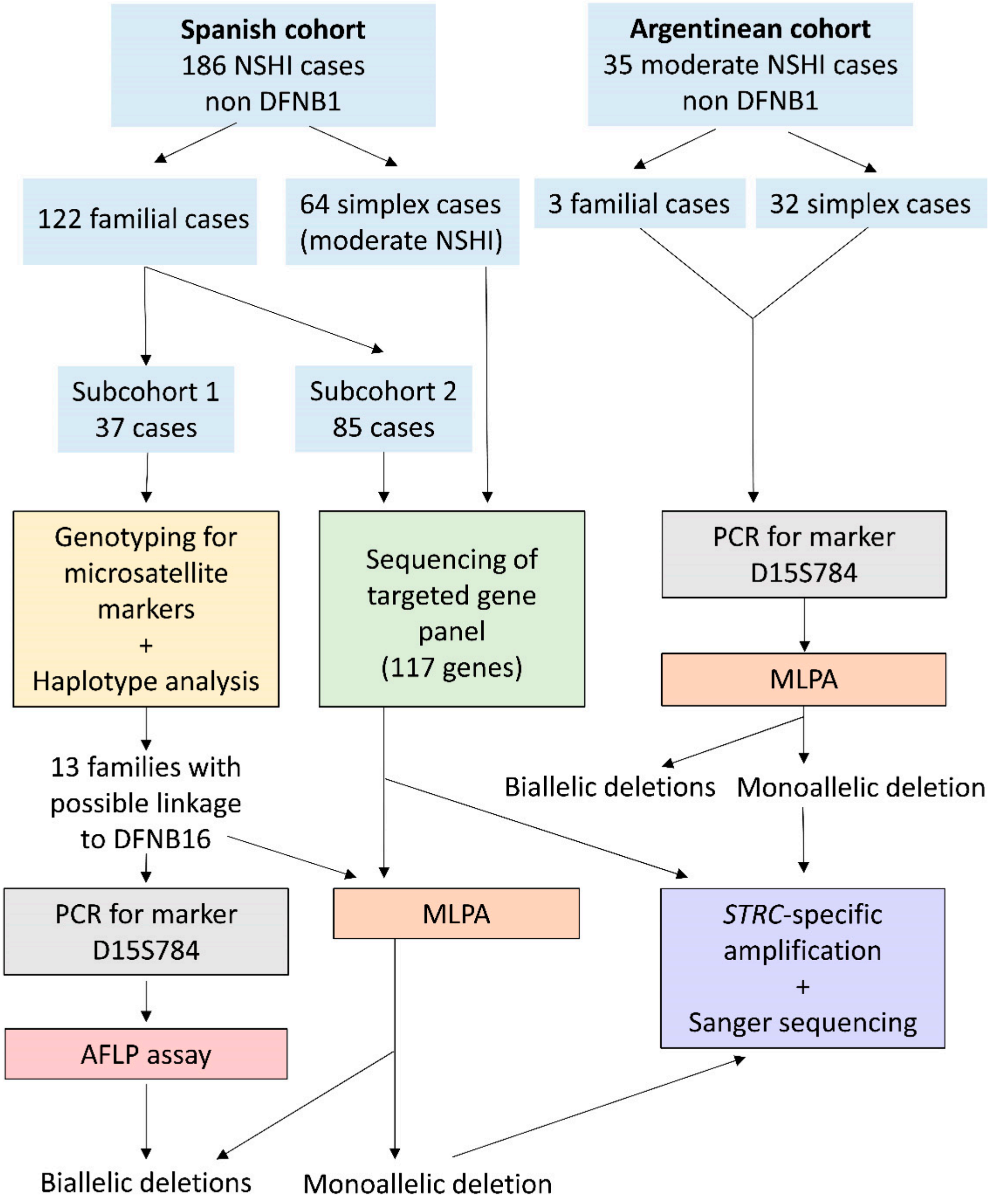

Figure S1. Diagram of the study setup.

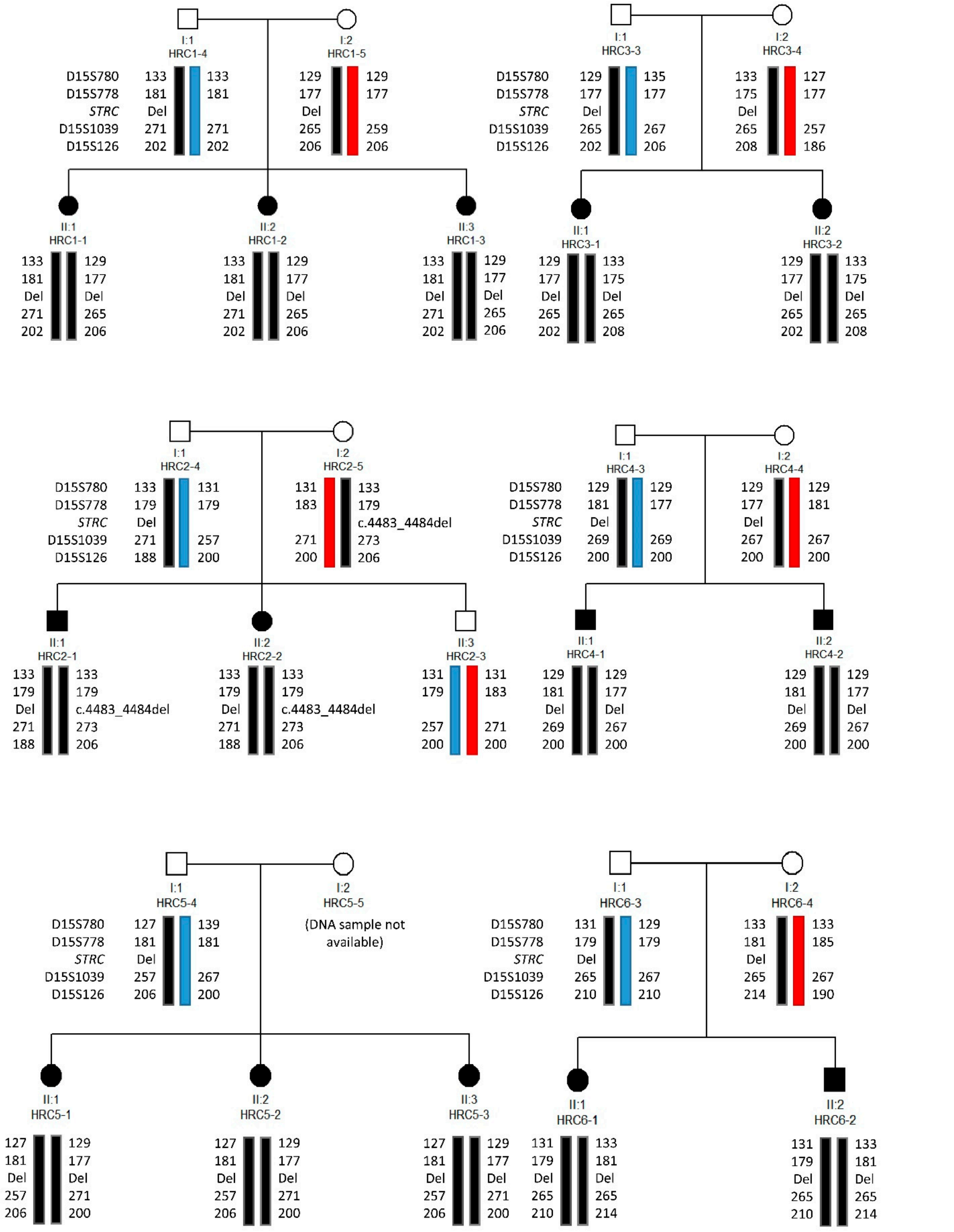

Figure S2. Pedigrees of the six families with biallelic pathogenic variants in the *STRC* gene for which haplotype analysis was performed. Haplotypes are indicated by vertical bars. In black, the haplotype associated with the disease. The four microsatellite markers that were genotyped and the position of the *STRC* gene are indicated on the left of the first subject in each family. Numbers indicate allele size, using as reference the genotype of CEPH subject 134702 (D15S780: 127/133; D15S778: 179/181; D15S1039: 267/267; D15S126: 188/202) [10, 11].

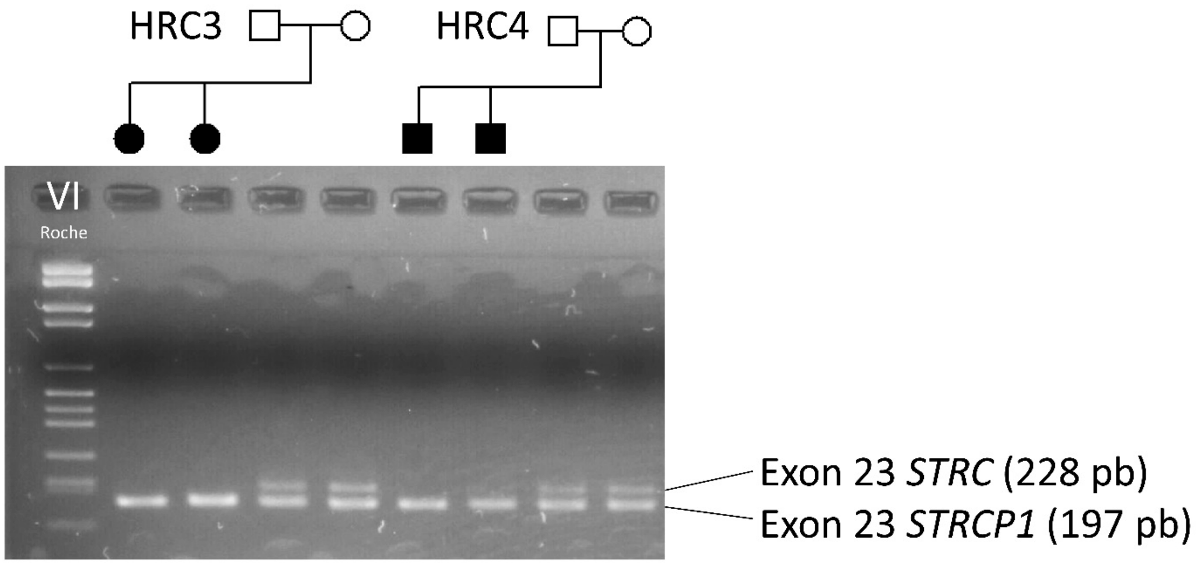

Figure S3. Example of AFLP test for the 3′ end of intron 22 in *STRC* and *STRCP1*. PCR products were resolved on a 2.5% agarose gel. Affected subjects of families HRC3 and HRC4 show only the band corresponding to *STRCP1*, whereas their parents show the two bands, corresponding to *STRC* and *STRCP1*, respectively.

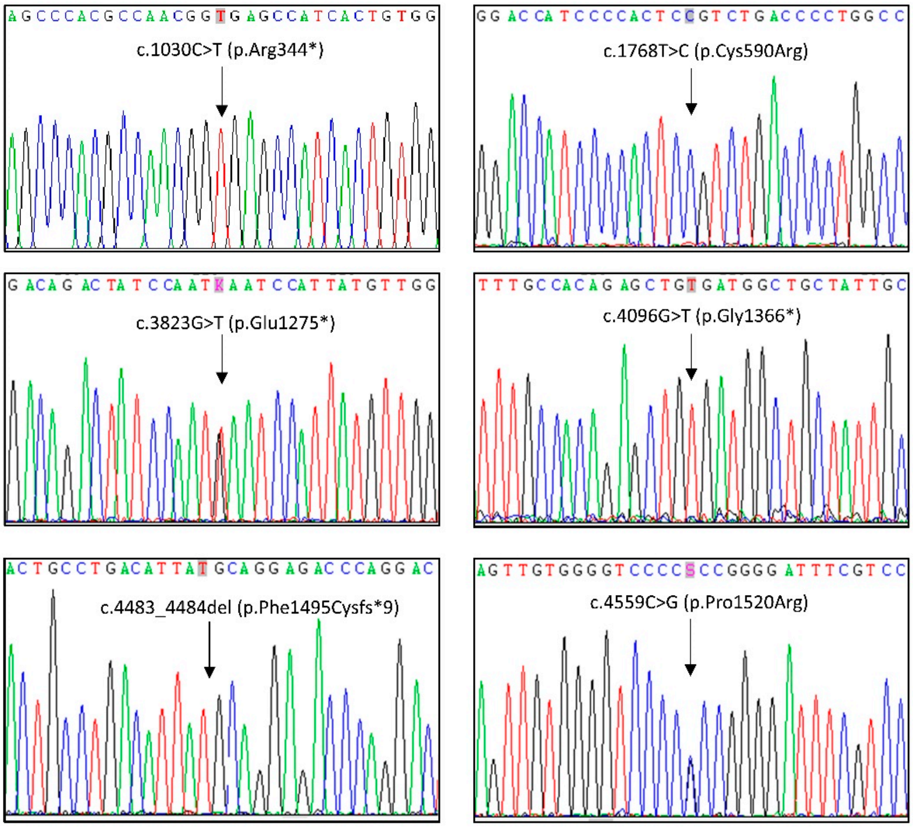

Figure S4. Electropherograms of the pathogenic SNVs in *STRC* that were found in this study.

```
                                             Cys590Arg
H.sapiens     ISRGGNDTCFLEGLLGPLLPSLPPLGPSPLCLTPGPFLLGMLSQLPRCQSSVPALAHPTR
B.taurus      ISRGGNDTCFLEGLLGPLLPSLPPLGPSPLCLAPGPFLLGMLSQLPRCQSSVPALAHPTR
M.musculus    ISRGGNDTCFLEGMLGPLLPSLPPLGPSPLCLAPGPFLLGMLSQLPRCQSSVPALAHPTR
M.domestica   VSRGGNDTCFLEGVLGPLLPPLPPLGPSPLCLAPGPFLLGMLSQLPRCQAAVPALSHPTR
               ************ ****** *********** ****************  **** ****
```

Figure S5. Alignment of estereocilin orthologous sequences from human and three other mammals in the stretch containing the residue that is affected by the p.(Cys590Arg) variant. Asterisks indicate identical residues across all sequences. Sequence accession numbers: *Homo sapiens* (NP_714544.1), *Bos taurus* (XP_024838009.1), *Mus musculus* (XP_006498797.1), *Monodelphis domestica* (XP_056671975.1). No orthologues were found in the databases for *Gallus gallus* (chicken), *Xenopus tropicalis* (clawed frog), *Danio rerio* (zebrafish), *Drosophila melanogaster* (fruit fly) and *Caenorhabditis elegans* (nematode).

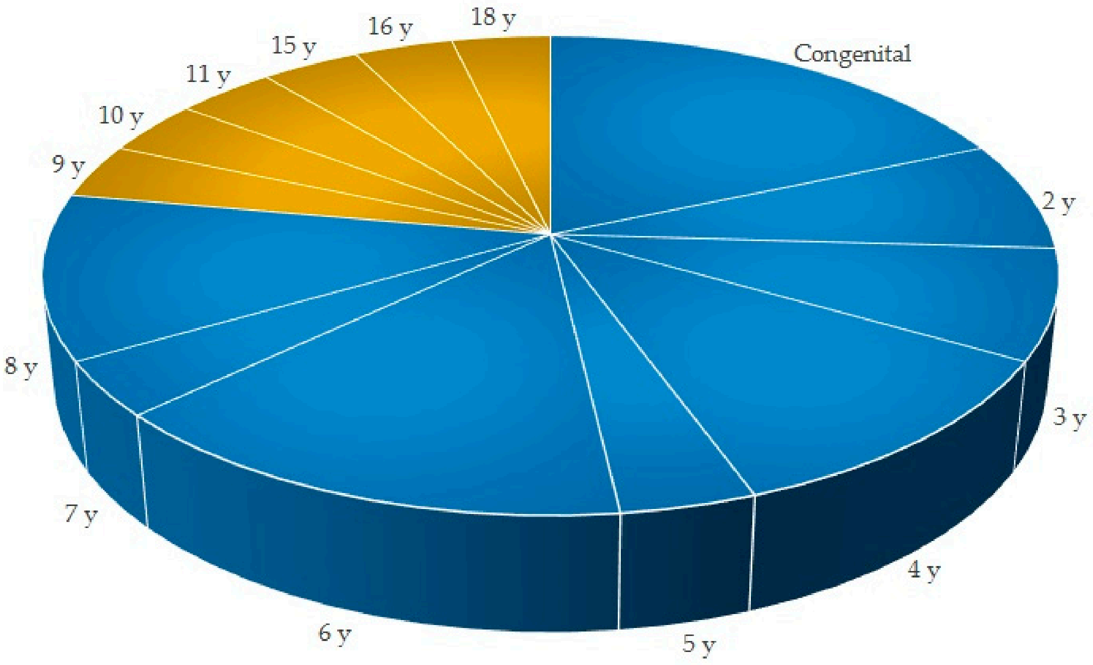

Figure S6. Age of onset of the hearing loss in 27 subjects with biallelic pathogenic variants in *STRC*. In blue, onset in early childhood (ages 0-8 years). In brown, apparently delayed onset (ages 9-18 years).

Table S1. Primer sequences for Sanger sequencing of the *STRC* gene.

**Amplicon**	**Upper primer (5′→3′)**	**Lower primer (5′→3′)**
**STRC_EX1**	TCCCCTATCATTAGTCCCTCTCACA	CACAAGTCTCTGCCTCTCTCCTTTG
**STRC_EX2F1**	GCTCAGGGGCAGGGTTGAG	GTCAGCAGCCAGGACACAGTCA
**STRC_EX2F2_EX3**	TGGACTTGTGGAGGTGCTGCT	CAGCTCTTCACCTCCAGATTTTCC
**STRC_EX4F1**	GCTTGCCATGCCCCTTGAG	GTCGCTCAGCCCACAGAGTCTC
**STRC_EX4F2**	CTGCTCCCACCCCCTACCA	GCGTGTGGGGTGAGCAAGA
**STRC_EX4F3**	TCTGACCCCTGGCCCCTTC	GCTTTCCCTCTGGCAGAGCAC
**STRC_EX5_EX6**	TGCAGGAGAGACAGAGAAGACAGCT	GAGATGCTCAGTATCCCTGCCATT
**STRC_EX7**	GCTTGCTTTAGTGGGAGGAGGAA	ACCGTGACAGGAGCAGGAGGA
**STRC_EX8_EX9**	AGGGCTACAGAGGGTCAGAGGAA	TGACAGCTGTGCCTTCCTCTCTC
**STRC_EX10**	GTGCCCCTGTGTAGTGGTGTAGTGT	AACAGGTAAGAATGGCCATGGAGT
**STRC_EX11**	TGGGAAATTCAGATGTGGGATTAGA	GCGTCCTCCTGCAGACAGAGTT
**STRC_EX12_EX13**	TTGCCTTAGGAACCCACTTAGGAC	GATGCCTTCCTCCCAACCAGA
**STRC_EX14**	GTGGCAAGGAGGATGAGATAATCAG	ACCAGAACTCTCAGGGCCACTG
**STRC_EX15**	CCCCATGGTTCCTTTTCTCTGA	GGCTAAGGGATAGGTAAAGAAGGTAGACTA
**STRC_EX16**	CACAGAGTGAGGAGGCAGGACTG	CAAAGGGAGGTGCTAGGAGAACG
**STRC_EX17_EX18**	ACAGGCTGTTGTATGTGTTGAATGAG	GGGAACCAGTCAATACATAGAGCAACT
**STRC_EX19**	CACTAAATTTGGTCTGGGGTCACAT	AGAGATTTCTCGGACTCCAAGAGGTAT
**STRC_EX20**	AGGGGGCTTGGTGTCTTGATC	GGAACAGGCTGCCACTGATG
**STRC_EX21**	GCATGTCCTCTTTTCCCCTTCC	GCTCACCAGTCCCCTCTATGCA
**STRC_EX22**	TGCAAAACAGAGACAGGACTTTGG	TTTGATACTCCCTGTACATCCTGCTG
**STRC_EX23**	TATTTCTCACAGTGCTCTATCCTCCAA	ATCTCTTGCTTCCCCCACCTCT
**STRC_EX24**	AATAAGCGTCTCTGTGTCTGATGCA	GATGATCTATAGGGCTGGGTCTGTG
**STRC_EX25**	TCCTCAGCCTTCCCCATGACT	AAATGAGTTCCCTTCCTCCATGG
**STRC_EX26**	TCTCATGACCACAGCCCCTTTC	CTAGCCCCTTATTTAAACACCCTCAG
**STRC_EX27_EX28**	AGGGCACTTTGGGAGTAGTTAGAGAA	GAGCCAGACAGCACCAAATTAGG
**STRC_EX29**	ACAGGCAGAGCGCTAATTTCCA	CGCCAGCTGATGACTCAAGATTC
